# An optimized approach for local de novo assembly of overlapping paired-end RAD reads from multiple individuals

**DOI:** 10.1098/rsos.171589

**Published:** 2018-02-28

**Authors:** Yu-Long Li, Dong-Xiu Xue, Bai-Dong Zhang, Jin-Xian Liu

**Affiliations:** 1 CAS Key Laboratory of Marine Ecology and Environmental Sciences, Institute of Oceanology, Chinese Academy of Sciences, 7 Nanhai Road, Qingdao 266071, Shandong, People's Republic of China; 2 Laboratory for Marine Ecology and Environmental Science, Qingdao National Laboratory for Marine Science and Technology, Qingdao 266071, People's Republic of China

**Keywords:** overlapping paired-end sequencing, RAD-seq, optimized assembly, pipeline software, optimal clustering

## Abstract

Restriction site-associated DNA (RAD) sequencing is revolutionizing studies in ecological, evolutionary and conservation genomics. However, the assembly of paired-end RAD reads with random-sheared ends is still challenging, especially for non-model species with high genetic variance. Here, we present an efficient optimized approach with a pipeline software, RADassembler, which makes full use of paired-end RAD reads with random-sheared ends from multiple individuals to assemble RAD contigs. RADassembler integrates the algorithms for choosing the optimal number of mismatches within and across individuals at the clustering stage, and then uses a two-step assembly approach at the assembly stage. RADassembler also uses data reduction and parallelization strategies to promote efficiency. Compared to other tools, both the assembly results based on simulation and real RAD datasets demonstrated that RADassembler could always assemble the appropriate number of contigs with high qualities, and more read pairs were properly mapped to the assembled contigs. This approach provides an optimal tool for dealing with the complexity in the assembly of paired-end RAD reads with random-sheared ends for non-model species in ecological, evolutionary and conservation studies. RADassembler is available at https://github.com/lyl8086/RADscripts.

## 1. Introduction

Recent developments of high-throughput sequencing techniques are revolutionizing studies of ecological, evolutionary and conservation genetics. Restriction site-associated DNA sequencing (RAD-seq) [1,2], which harnesses the massive throughput of next-generation sequencing, enables low-cost discovery and genotyping of thousands of genetic markers in both model and non-model species [3,4]. Illumina paired-end (PE) sequencing techniques make the original RAD (RPE) [5,6] more attractive for de novo studies. The first reads begin at the restriction enzyme cut site while the second reads are staggered over a local genomic region of usually several hundred base pairs. Furthermore, the overlapping RPE reads of each RAD locus could be individually assembled into one contig with the enzyme cut site at one end. The assembled contigs can provide more sequences information for blast annotations and the removal of paralogues [4,6,7]. In addition, RPE reads can also be used to remove polymerase chain reaction (PCR) duplicates, which will improve downstream genotyping accuracy, and the overlapping reads can further improve genotyping accuracy towards the ends of the reads [4].

To increase sequence coverage for RAD contigs assembly, it is a standard practice to pool multiple individuals' reads, which might introduce assembly complexity especially for non-model species with little knowledge of the genomic background [8,9]. Assembly software is challenged by repeats, sequencing errors, polymorphisms in the target and the computational complexity of large data volumes [10]. The polymorphisms among different individuals also complicate the assembly, and this could be more challenging particularly for species with high genetic variance. The assembly for RPE reads is more difficult compared to other RAD approaches that produce RAD loci of fixed length (flRAD), such as ddRAD [11]. PE ddRAD is much easier to assemble, because both the paired reads start at the restriction enzyme cut sites with fixed read length of uniform coverage of depth, and the reads could be easily stacked up. However, RPE is more difficult to assemble, as the second reads are staggered because of sonication and size selection, thus their coverage is non-uniform. In addition, there is huge difference of depth between the first reads and the second reads, which makes the assembly of RPE reads more challenging.

Previous studies have assembled RPE reads into contigs using different assembly tools [5,8,12], such as the de Bruijn Graph (DBG) based software Velvet [13] and the Overlap-Layout-Consensus (OLC) based software CAP3 [14] and LOCAS [15]. Davey *et al.* [9] demonstrated that VelvetOptimiser was the best assembly tool for RAD data by comparing nine assembly tools. However, Hohenlohe *et al.* [8] found CAP3 performed much better than Velvet. The results of Hohenlohe *et al.* showed that most reads of a locus could be each assembled into one contig by using CAP3, while Velvet failed to connect the overlapping PE reads at many loci. Possible causes of the conflicting results between the two studies might be attributed to the fact that Davey *et al*. did not use the overlapping RPE library preparation protocol and they only used the second reads for assembly, and therefore the information for the first reads was lost. There are several software for the assembly of RAD data supporting PE reads, such as Stacks [16,17], Rainbow [18], pyRAD [19] and dDocent [20]. However, many of these tools cannot directly and fully support RPE datasets with staggered PE reads. There are many studies which did not make full use of RPE reads either for assembly or single nucleotide polymorphism (SNP) discovery due to the lack of software or approaches that are specially optimized for RPE assembly. Therefore, an easy-to-use software as well as an approach specially optimized for the assembly of RPE reads is urgently needed. Here, we present an optimized assembly approach with a pipeline software, RADassembler, to deal with the complexity of RAD assembly, which could take full advantage of the overlapping RPE reads.

The goals of this study are to: (a) present an optimized approach with the pipeline software RADassembler for local de novo assembly of the overlapping RPE reads from multiple individuals and (b) compare the performances of RADassembler with the original Stacks, Rainbow, and dDocent on both simulation and real RPE datasets.

## 2. Material and methods

By making full use of the features of RPE reads, we can firstly cluster the first reads (the forward reads with enzyme cut sites) into RAD loci based on the sequence similarity, then group the read pairs of each locus accordingly and perform the local de novo assembly. The pipeline software RADassembler, written in Bash and Perl, mainly uses Stacks and CAP3 to perform the local de novo assembly of the RPE reads. Specifically, Stacks is used for clustering, and CAP3 is used for assembly. We chose Stacks (version 1.48) for clustering due to its popularity in analysing RAD-seq data in prior studies.

### 2.1. Choosing the optimal similarity thresholds for clustering

As the similarity thresholds (the number of mismatches) for clustering are critical for the downstream analysis, we adopted a protocol from Ilut *et al.* [21] for optimal similarity threshold selection within individuals. Two main components of Stacks were used for the selection of optimal similarity thresholds, ustacks and cstacks. Data from each individual were grouped into loci by ustacks, and loci were grouped together across individuals by cstacks. RADassembler would run ustacks of Stacks using a set of mismatches (e.g. from 1 to 10) using a single individual. The optimal number of mismatches within individual was chosen to maximize the number of clusters with two haplotypes (alleles) and simultaneously minimize the number of clusters with one haplotype (allele). A novel method for choosing the similarity threshold across individuals (cstacks) was also introduced: RADassembler would run cstacks of Stacks using a set of mismatches (e.g. from 1 to 10) on a subset of data (e.g. randomly select several individuals from each population). The optimal number of mismatches across individuals was chosen at the point of inflection, such that the number of incremental loci for each merging individual using different mismatches changed little. All the above parameters can be set by users.

### 2.2. De novo assembly of RAD contigs

After choosing the optimal number of mismatches within and across individuals, the first reads were sent to Stacks for clustering. A minimum depth of 5 was set to create a stack, and the number of mismatches allowed between stacks was set to the optimum to maintain the true alleles from paralogues. Deleveraging and removal algorithms of Stacks were turned on to resolve over merged loci and to filter out highly repetitive, likely paralogous loci. When building the catalogue, the number of mismatches allowed between loci across individuals was set to the optimum to attempt to merge loci together. Finally, only the second reads of each RAD locus from multiple individuals were collected into separate fasta files by using a modified version of ‘sort_read_pairs.pl' of Stacks. RADassembler used data reduction techniques to select a certain number of reads (maximum of 400 and minimum of 10, set by users) for assembly.

**Figure 1.**
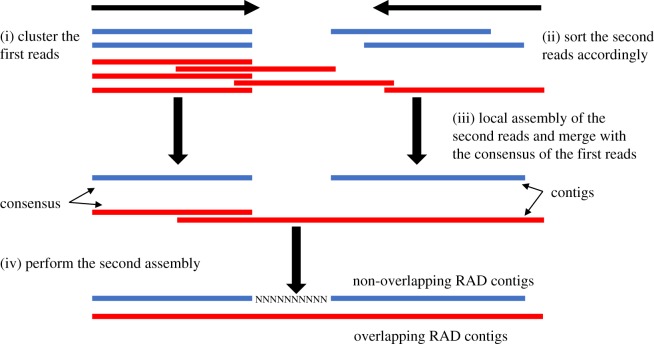
Flow chart for the two-step assembly approach on RPE reads. (i) The first reads (the forward reads with enzyme cut sites) were clustered. (ii) The second reads (the reverse reads with random-sheared ends) were sorted into separated files accordingly (each locus represented by different colours contained reads from multiple individuals). Reads were assembled by a two-step assembly strategy: (iii) first step, the second reads were locally assembled into contigs and merged with the corresponded consensus sequences of the first reads; (iv) second step, the merged files were locally assembled again into the final RAD contigs. If the contigs of the second reads do not overlap with the consensus sequences, ten ‘N’ will be padded (locus in blue).

To reduce the complexity for assembly, we present here a two-step assembly approach implemented in RADassembler (figure 1). Firstly, the second reads (the reverse reads) with random-sheared ends from multiple individuals corresponding to each RAD locus were sent to CAP3 to assemble separately, and the resulting contigs of each locus were then merged with the corresponding consensus sequence of the first reads from Stacks catalogue into one file. Secondly, each merged file was then locally assembled again into the final RAD contigs using CAP3. In the second step, if the contigs from the first step did not overlap with the consensus sequences, they would be concatenated by ten ‘N'. The assembly approach was parallelized to achieve the maximum efficiency. RADassembler used parameters specifically optimized for short reads assembly following the manual of CAP3 (see electronic supplementary material for the details of parameters).

### 2.3. RADassembler on simulation data

**Figure 2.**
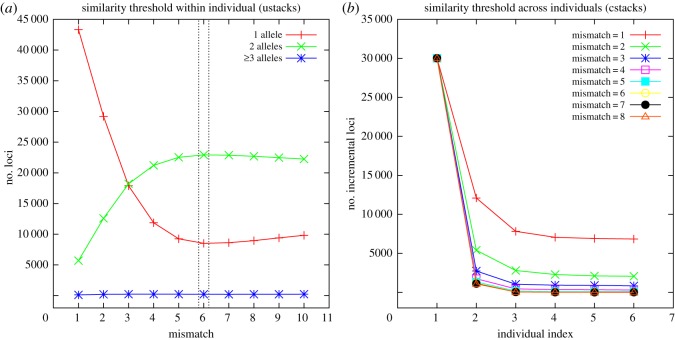
The selection of the optimal number of mismatches within (*a*) and across (*b*) individuals on simulation datasets. Reads from each individual were grouped into loci by ustacks, and loci were grouped together across individuals by cstacks to build the catalogue. The optimal number of mismatches within individual (ustacks) was chosen to maximize the number of loci (*Y*-axis on the left) with two alleles and simultaneously minimize the number of loci with one allele. In this case, six mismatches should be an appropriate value for ustacks. For cstacks, the optimal number of mismatches across individuals was chosen at the point of inflection, such that the number of incremental loci (*Y*-axis on the right) for each merging individual (*X*-axis on the right) using different mismatch thresholds (represents by different line types) changed little. In this case, four mismatches should be an appropriate value for cstacks.

To evaluate the performance of RADassembler, we simulated 12 individuals with high levels of heterozygosity (0.02) on reference genome of the Genome Reference Consortium Zebrafish Build 11 (GRCz11, NCBI accession: GCF_000002035.6) digested with the enzyme SbfI. Only the primary assembly on 25 chromosomes of GRCz11 were retained for *in silico* digest. By using ‘ezmsim', a modified version of ‘wgsim' [22] from Rainbow, PE reads of length 125 bp were simulated from a range of insert size libraries initiated from 200 bp and elongation of 10 steps, with each step extends 50 bp. Mean depth of the PE reads was set to 10 for each step, and a sequencing error rate of 0.01 was randomly introduced according to a common error rate of approximately 0.1–1 × 10^−2^ for Illumina sequencing machines [23]. So the expected coverage for each simulated RAD locus is 700 bp, and SNPs were random across all individuals. After checking the optimal number of similarity thresholds (see Results and figure 2), the number of mismatches within individual (ustacks) was set to 6, and the number of mismatches across individuals (cstacks) was set to 4. All the simulation and subsequent analysis were performed on a workstation with 20 CPUs (2.30 GHz) and 256 GB memory; 30 threads were used when parallelization was available.

### 2.4. RADassembler on real data

**Figure 3.**
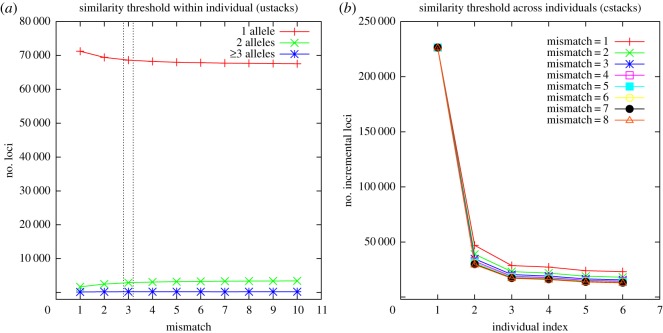
The selection of the optimal number of mismatches within (*a*) and across (*b*) individuals on real datasets (*L. polyactis*). The optimal number of mismatches within individual should be 3 (*a*), and optimal number of mismatches across individuals should be 3 (*b*), although liberal values might be more appropriate.

Overlapping RPE reads of 24 individuals for the small yellow croaker *Larimichthys polyactis* from Zhang *et al.* [24] were selected as a real dataset, with approximate insert sizes from 200 to 600 bp. Raw reads were firstly processed by cutadapt [25] to remove potential adaptors, then were passed to process_radtags of Stacks to drop low-quality read pairs with a window size of 0.1 and a score limit of 13. Only read pairs containing enzyme cut sites were retained. In addition, PCR duplicates were removed by clone_filter of Stacks. The final retained reads from the 24 individuals were sent to RADassembler for optimized RAD contigs assembly. The numbers of mismatches were set to 3 (ustacks) and 3 (cstacks) following the optimal similarity thresholds choosing method (see Results and figure 3). The assembled contigs were adaptor removed using cutadapt and a minimum contigs length of 125 bp was also required.

### 2.5. Comparisons of the performance to other tools

We compared the assembly performance of RADassembler with three other popular tools that supported RPE reads, including the original Stacks (version 1.48), Rainbow (version 2.04) and dDocent (version 2.2.20). The performances of assembly on both simulation and real datasets were compared. Parameters in the original Stacks were identical to that used in RADassembler, except that all the read pairs from multiple individuals for each locus were extracted by using a modified version of ‘sort_read_pairs.pl’, and then were sent to the wrapper ‘exec_velvet.pl’ provided by Stacks to assemble contigs. This wrapper will run Velvet on each locus and collect the sequences into the final contigs; a minimum contig length of 125 bp was required. Rainbow is an ultra-fast and memory efficient solution for clustering and assembling short reads produced by RAD-seq. Rainbow includes three steps in assembling RAD contigs: clustering, dividing and merging (assembly). Parameters in Rainbow were set according to those used in RADassembler and dDocent, which were adjusted for multiple individuals. dDocent is an analysis pipeline that uses data reduction techniques and other stand-alone software packages to perform quality filtering, de novo assembly of RAD loci, read mapping, SNP calling and data filtering. Cut-off values for coverage of reads in the first assembly step of dDocent were set to 5 (within individual) and 2 (across individuals), respectively; similarity threshold for the last reference clustering was set to the optimal value used in RADassembler clustering stage. All the detailed parameters used in the above programs are presented in the electronic supplementary material.

We evaluated the performances of the assembly of different tools using the commonly used statistics, including N50, mean contig length and total contig length (coverage). Besides, for simulation data the assembled contigs were also mapped to the original reference genome using the local BLAST+ [26] program, the mean identity and coverage were calculated. For real datasets, as no reference genome was currently available for *L. polyactis*, the reference genome (NCBI accession: GCF_000972845.1) of the congener (*Larimichthys crocea*) was selected for blast mappings. Furthermore, read pairs were also mapped back to the assembled contigs by using BWA 0.7.15 [27] to check the number of mapped reads and properly mapped reads. The properly mapped reads were those with the forward read and the reverse read mapped on the same contig (loci) and with right orientation as well as proper insert size, which was identified by SAM flags given by the aligner. For simplification and consistency, only the read pairs used for the assembly in Stacks were used for all the reads mappings, which would represent a comprehensive subset of the raw input reads. The ‘mem' algorithm [28] in BWA was used for mapping, and the parameters were set to default. Mapping statistics were calculated by Samtools 1.6 [22].

## 3. Results

### 3.1. Comparisons of RAD contigs assembly on simulation data

**Figure 4.**
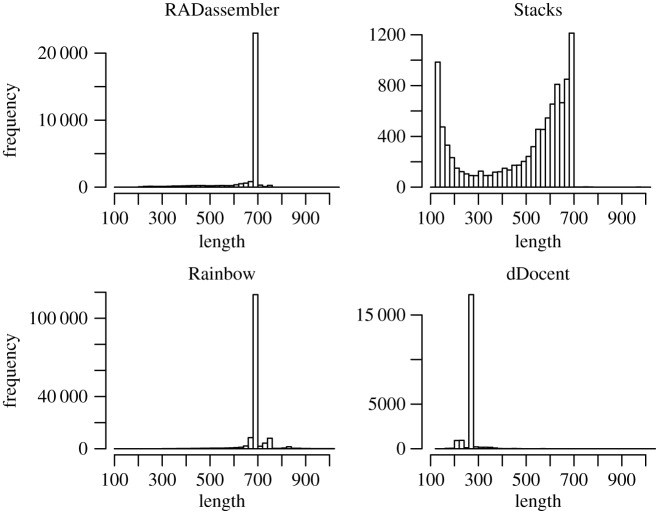
Length distribution of contigs assembled by the four tools on simulation datasets. Program versions: Stacks 1.48, Rainbow 2.04, dDocent 2.2.20.

Using *in silico* digest, there were 29 242 cut sites of SbfI on the main assembly of the 25 chromosomes of GRCz11. Thus, an expected approximate coverage of 20 469 400 bp RAD library for each individual was generated, which covered approximately 1.52% of the genome. Using a set of mismatches (from 1 to 10 for ustacks, from 1 to 8 for cstacks) to cluster the first reads from preliminary runs, the optimal mismatches within individual (ustacks) was set to 6, and the optimal number of mismatches across individuals (cstacks) was set to 4 (figure 2). RADassembler exported a total of 29 533 loci for assembly, all of which were successfully assembled. The assembled contigs was with an N50 of 698 bp, mean contig length of 661 bp and a total coverage of 19 633 933 bp (table 1). Length distribution for the assembled contigs is presented in figure 4.

**Table 1.** Assembly statistics of the four tools on simulation datasets. Comparison statistics including (from left to right): number of clusters (loci) assembled, number of clusters that mapped to the reference genome (Identical Clusters), N50 (bp), mean contig length (Mean, bp), total coverage (Total Cov, bp), identical bases to the reference genome (Identical Cov, bp), identical bases to the reference genome in proportion of the total coverage (Cov Ratio), mean identity of those mapped to the reference genome (Mean Identity), total mapping rate of the read pairs (Total Mapped), proper mapping rate of the read pairs (Proper Paired).

simulation datasets	no. clusters	Identical Clusters	N50	Mean	Total Cov	Identical Cov	Cov Ratio (%)	Mean Identity (%)	Total Mapped (%)	Proper Paired (%)
RADassembler	29 533	29 521	698	661	19 633 933	19 401 812	98.82	98.78	99.94	98.60
Stacks	8717	8491	617	480	4 889 191	4 774 282	97.65	98.25	37.00	11.12
Rainbow	154 410	154 375	696	695	107 402 013	102 362 273	95.31	97.72	99.97	95.05
dDocent	20 248	20 222	262	261	5 297 706	4 884 498	92.20	98.67	77.66	36.62

Compared to the other three tools, RADassembler identified the most appropriate number of clusters (loci), and the assembled contigs generally showed high qualities (table 1). By mapping the contigs to the reference genome, 99.96% of the clusters (assembled contigs) were mapped to the reference, with a mean identity of 98.78%. RADassembler showed the highest coverage ratio and proper mapping rate, with 98.60% of the reads properly mapped. Stacks and dDocent assembled many contigs of short length, which were not in accordance with the expectation (should be around the maximum insert size, 700 bp). Stacks (Velvet) failed to assemble most loci, though it recovered the appropriate number of loci in the clustering stage. The original Stacks assembled only 8717 loci, and most of the reads could not be properly mapped back (only 11.12% were properly mapped), which might suggest that Velvet was inappropriate for the assembly of RPE reads. Rainbow assembled much more loci (154 410) than the other tools, which was not in accordance with the expectation, suggesting the existence of many redundant loci. dDocent assembled 20 248 loci with an N50 of only 262 bp. When mapping back the read pairs, only 36.62% of the read pairs were properly mapped to the assembled contigs of dDocent. Although dDocent was the most time-efficient one among the four tools (see electronic supplementary material for benchmark details), RADassembler was still more efficient than the original Stacks and Rainbow. From a comprehensive perspective, RADassembler was the best performing tool among the four and comparison details are presented in table 1.

### 3.2. Comparisons of RAD contigs assembly on real data

After quality filtering, a total of 62 960 475 read pairs were retained for the 24 individuals of *L. polyactis*, with a mean read pairs of 2 623 353 per individual. Using preliminary runs to check the optimal similarity thresholds, the number of mismatches within individual was set to 3, and the number of mismatches across individuals was set to 3 (figure 3). RADassembler exported a total of 303 929 loci for assembly and all of these were successfully assembled. The assembled contigs, with an N50 of 539 bp, mean contig length of 511 bp and a total coverage of 157 941 578 bp, also demonstrated high qualities (table 2). Most of the read pairs (98.98%) were mapped to the contigs, and 95.99% of these were properly mapped. When mapping the assembled contigs to the reference genome of *L. crocea*, 98.33% of the assembled contigs were mapped to the reference with a mean identity of 95.85%.

**Table 2.** Assembly statistics of the four tools on real datasets of *L. polyactis*. The parameters of comparisons were the same as those used in the simulation datasets.

real datasets	no. clusters	Identical Clusters	N50	Mean	Total Cov	Identical Cov	Cov Ratio (%)	Mean Identity (%)	Total Mapped (%)	Proper Paired (%)
RADassembler	303 929	298 866	539	511	157 941 578	144 559 665	91.53	95.85	98.98	95.99
Stacks	460 525	451 478	344	282	181 151 234	164 990 228	91.08	96.27	87.89	49.16
Rainbow	330 584	325 298	579	550	182 080 648	160 647 819	88.23	95.47	92.34	85.47
dDocent	183 763	179 928	262	271	49 820 676	43 255 514	86.82	96.70	83.44	61.62

**Figure 5.**
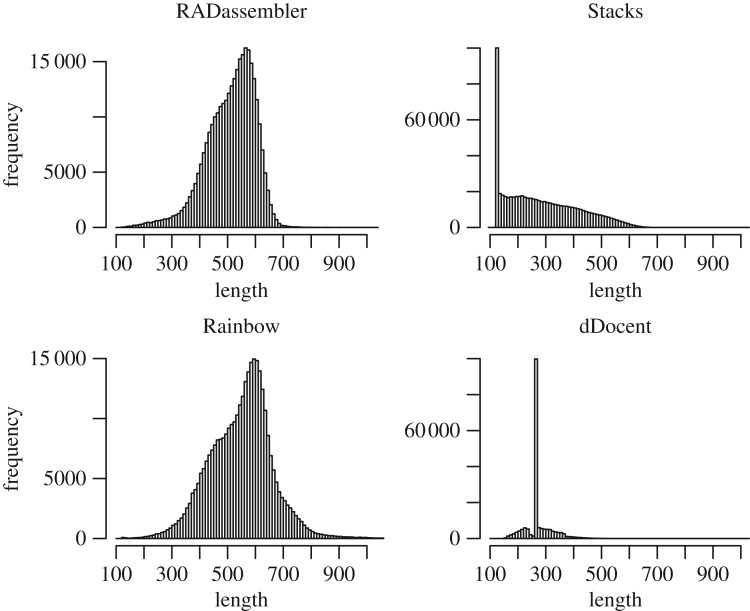
Length distribution of contigs assembled by the four tools on real datasets (*L. polyactis*). Program versions: Stacks 1.48, Rainbow 2.04, dDocent 2.2.20.

RADassembler was also more competent than the other three tools on the real datasets (table 2). It always showed the highest proper mapping rate, and the length of contigs conformed to the expected size (figure 5). Similar to their performances on simulation datasets, Stacks (Velvet) and dDocent performed poorly in recovering the appropriate contigs size on the real datasets (figure 5), with many of them were short ones. The original Stacks (Velvet) and Rainbow assembled more clusters (loci), and the total coverage was 181 151 234 bp and 182 080 648 bp, respectively. However, a large proportion of the read pairs could not be properly mapped. For the original Stacks, 87.89% of reads were mapped, but only 49.16% of these were properly mapped. However, Rainbow performed better than Stacks on the real datasets and itself on the simulation datasets. The size of the assembled contigs by Rainbow was also in accordance with the expected insert size. Moreover, the total and proper mapping rate is 92.34% and 85.47%, respectively, but is still not as good as RADassembler. dDocent assembled 183 763 clusters, and size of most of the assembled contigs was small, which was consistent with its performance on the simulation datasets. Most of the contigs assembled by dDocent were around 260 bp, which was the length of the forward read (125 bp) and the reverse read (125 bp) plus ten ‘N', suggesting its failure in the assembly of the second reads with randomly sheared ends (figure 5). The details of comparison of performance of the tools are presented in table 2.

## 4. Discussion

Several analysis tools have been released and widely applied to help researchers to deal with RAD-seq data. However, previous studies based on RPE have only used either the first reads [29,30] for SNP calling and downstream population genetic analysis, or only the second reads for contigs assembly [6,31,32], and the information for the other reads was wasted then. Although most of these tools support PE reads, many of them do not directly support RPE reads with random-sheared ends. Many studies have not taken full advantage of RPE read pairs for both assembly and SNP calling. The main constraints here may be the highly uneven coverage depth of the read pairs and the generally low depth of the second reads, as shown in Davey *et al.* [9]. However, RADassembler helped in reducing the complexity of RPE assembly, and the results presented in this study demonstrated its high promise and wide applicability.

RADassembler offered two advantages in its assembly for RPE reads: (i) it used methods to choose the optimal similarity thresholds within and across individuals and (ii) it used a two-step assembly approach to efficiently reduce the assembly complexity. Similarity threshold selection is critical for the downstream analysis. Stringent thresholds will cause over-splitting, which creates false homozygosity, and liberal thresholds will cause under-splitting, which creates false heterozygosity [21,33]. Incorrect similarity thresholds affect the inferences of the level of variation in the downstream population genetic and phylogeographic estimates [33]. RADassembler could efficiently identify the optimal threshold within and across individuals without the prior knowledge of heterozygosity. As a pipeline software, dDocent also includes a two-step strategy in the assembly of RAD reads, but the rationale of which is quite different from RADassembler. dDocent was originally designed and optimized for flRAD datasets [20], although its current version also supports RPE datasets. At the first step of assembly, dDocent uses the concatenated PE reads (only the first reads were used for RPE) to count the occurrences of unique reads, then users can choose a cut-off level of coverage for reads to be used for assembly. The choice for a cut-off of unique reads within individual is similar to that in ustacks (the parameter of minimum depth of coverage required to create a stack) of Stacks. The remaining concatenated reads are then divided back into read pairs, clustered and locally assembled by Rainbow (in the current version of dDocent, CD-HIT [34,35] is used for clustering). At last, the assembled contigs are clustered based on the overall sequence similarity using CD-HIT. By contrast, RADassembler only uses the second reads of each locus for local assembly in its first assembly step. The assembled contigs for the second reads are then merged (either assembled or padded by ten ‘N') with the corresponding consensus sequences of the first reads. The output reference contigs of dDocent represent only a subset of the total genomic information content of the raw input [20], which might be the cause of its lower proper mapping rate in the results. However, RADassembler will assemble more comprehensive information for a de novo assembly of RAD loci. The comprehensive RAD reference is useful for downstream annotations and will increase the chance of discovering individual level polymorphisms.

RADassembler also supports multi-threading, and it includes a data reduction step before assembly. Users can choose a cut-off level of coverage to restrict the minimum and maximum number of reads for each locus used in assembly. Thus, RADassembler achieved a better running efficiency compared to the original Stacks and Rainbow. Rainbow includes a dividing step after the first clustering to distinguish sequencing errors from heterozygote or variants between repetitive sequences [18]. While this step worked perfectly for data of a single individual, it performed not so well in pooled data from multiple individuals, especially in species with high polymorphisms, as shown in the simulation datasets. Rainbow might be inappropriate for the assembly of RPE datasets from multiple individuals with high heterozygotes, though the parameters need further optimizations. RADassembler uses Stacks for better clustering and it is more appropriate in dealing with polymorphisms among multiple individuals. Stacks mainly uses two steps for de novo assembly of loci, ustacks for clustering within individuals and cstacks for constructing catalogue across individuals. The original Stacks uses DBG-based assembler Velvet to assemble contigs only for the second reads of RPE reads. By modifying the program to include the first reads, however, Velvet did not perform well and failed to connect overlapping RPE reads at many loci. Similar results were also observed in Hohenlohe *et al.* [8]. Both the OLC-based assemblers CAP3 (used in RADassembler) and Rainbow assembled the appropriate size of contigs, suggesting their advantages over DBG-based assemblers in the assembly of RPE reads.

There are two categories of widely used NGS assemblers, which are based on either the OLC methods or the DBG methods [10]. The OLC methods rely on an overlap graph involving three phases: overlap, layout and consensus [36]. The OLC-based assemblers perform pairwise alignments (which is computationally expensive) to discover overlaps and the length of overlaps are not required to be uniform. The DBG methods rely on k-mer graph, which use fixed-length subsequence (k-mer) as its nodes and overlaps between consecutive k-mer as its edges. K-mer graph methods do not require all-against-all overlap discovery [10], thus might lose some true overlaps, but have advantages in efficiency of assembly for high-throughput short reads. The k-mer graph-based assembler has been applied on RAD data in many studies, such as those using Velvet [32,37] and VelvetOptimiser [6,9]. However, DBG-based assemblers did not perform well in the presented study, as well as in Hohenlohe *et al.* [8]. The general problem may be due to the highly uneven sequence coverage of depth expected in each locus for the RPE datasets [8], which makes it hard for Velvet to correctly assemble contigs. Indeed, Velvet is confounded by non-uniform coverage of the target sequences, as it uses coverage-based heuristics to distinguish putative unique regions from putative repetitive regions [38]. Nonetheless, compared to overlap graphs, k-mer graphs are more sensitive to repeats and sequencing errors [10], suggesting that k-mer graph based tools (such as Velvet) might be less powerful for assembly of pooled reads from multi-individuals. The polymorphisms among individuals will also complicate the assembly, particularly for k-mer graph methods. The OLC methods performed much better though a bit more computationally expensive, but still affordable after data reduction and parallelization. Additionally, RADassembler uses a two-step strategy to further reduce the complexity of RPE assembly. This strategy offers two advantages: firstly, it reduces the complexity of reads from multiple individuals as well as the calculation demands by using consensus sequences of the first reads and data reduction techniques (randomly select a subset of reads); secondly, it makes the depth in each assembly step uniform. At the same time, it is also crucial for researchers to vary the parameters to optimize the assembly. One solution is to estimate assembly parameters for each locus [9], and use a hybrid assembly strategy (use both OLC and DBG assemblers). However, this would cause severe computational demands. Our approach presented here provides a good tool for dealing with the complexity of RAD assembly, particularly for assembly of RPE reads from multiple individuals with high genetic variation.

RAD contigs are attractive for the detection and annotation of loci of interest (e.g. outliers). The assembled contigs hold higher probabilities to hit the database than that of the single-end consensus sequences. These annotations are important for population genomic and conservation genetic applications. In addition, RAD contigs provide more chances for outlier detection. The longer continuous sequences are expected to contain more SNPs that might be relevant to local adaptations. The assembled RAD contigs also provide sufficient flanking sequences for the design of primers or arrays that could be further used to perform functional verifications or studies of adaptive evolution based on more samples.

In the present study, we provided an optimized approach with the pipeline software RADassembler to deal with the assembly complexity for RPE reads from multiple individuals. The results on both simulation and real datasets suggested its high accuracy and efficiency. RADassembler included the protocols for choosing the optimal similarity thresholds, data reduction techniques as well as a two-step assembly approach to reduce the assembly complexity for RPE reads. RADassembler could provide an optimal tool for dealing with the complexity of RAD assembly for non-model species in ecological, evolutionary and conservation studies, especially for species with high polymorphisms.

## Supplementary Material

Supporting information for the optimized RAD assembly

## Data accessibility

All the scripts and software used for data analysis are available at GitHub: https://github.com/lyl8086/RADscripts.
