# Supplementary material for: An optimized approach for local de novo assembly of overlapping paired-end RAD reads from multiple individuals
Source: R Soc Open Sci. 2018 Feb 28;5(2):171589. doi: 10.1098/rsos.171589 (PMC5830760; doi:10.1098/rsos.171589)
Supplement: Supporting information for the optimized RAD assembly [file rsos171589supp1.docx]

**Royal Society Open Science**

Electronic Supplementary Material

Title: An optimized approach for local de novo assembly of overlapping paired-end RAD reads from multiple individuals

Author: Yu-Long Li; Dong-Xiu Xue; Bai-Dong Zhang; Jin-Xian Liu

Contact: Jin-Xian Liu, [jinxianliu@gmail.com](mailto:jinxianliu@gmail.com) or Yu-Long Li, [liyulong12@mails.ucas.ac.cn](mailto:liyulong12@mails.ucas.ac.cn)

1. Parameters used in each program.

| Simulation datasets | Main parameters |
| --- | --- |
| RADassembler | main:-m 6 –n 4 –M 5 –D 10:400 –t 30  CAP3: -r 0 -i 30 -j 31 -o 18 -s 300 -p 85 |
| Stacks 1.48 | ustacks: -M 6 –m 5 –d –p 30  cstacks: -n 4 –p 30  sstacks: -p 30 |
| Rainbow 2.04 | cluster: -m 10  div: -f 0.5 –k 10  merge: -a -f 0.85 -r 10 -R 400 -N500 |
| dDocent 2.2.20 | default, except that the cut-off was set to 5 and 2, clustering similarity was set to 0.92 |

| Real datasets | Main parameters |
| --- | --- |
| RADassembler | main:-m 3 –n 3 –M 5 –D 10:400 –t 30  CAP3: -r 0 -i 30 -j 31 -o 18 -s 300 -p 85 |
| Stacks 1.48 | ustacks: -M 3 –m 5 –d –p 30  cstacks: -n 3 –p 30  sstacks: -p 30 |
| Rainbow 2.04 | cluster: -m 6  div: -f 0.5 –k 10  merge: -a -f 0.85 -r 10 -R 400 -N500 |
| dDocent 2.2.20 | default, except that the cut-off was set to 5 and 2, clustering similarity was set to 0.95 |

2. Running time for each program on simulation and real datasets. The benchmarks were performed on a workstation with Intel E5-2650v3*2 CPUs, 30 threads were used if available.

| Simulation datasets | No. of clusters assembled | Wall time | CPU time | System time |
| --- | --- | --- | --- | --- |
| RADassembler | 29,533 | 94m1.725s | 1471m38.776s | 19m44.955s |
| Stacks 1.48 | 8,717 | 215m34.308s | 2206m22.212s | 508m23.815s |
| Rainbow 2.04 | 154,410 | 198m44.698s | 197m32.195s | 2m37.072s |
| dDocent 2.2.20 | 20,248 | 5m23.555s | 14m36.104s | 1m7.869s |

| Real datasets | No. of clusters assembled | Wall time | CPU time | System time |
| --- | --- | --- | --- | --- |
| RADassembler | 303,929 | 376m52.009s | 2663m56.514s | 681m48.615s |
| Stacks 1.48 | 460,525 | 1976m38.789s | 28799m22.487s | 2153m33.960s |
| Rainbow 2.04 | 330,584 | 1062m19.262s | 1042m22.052s | 22m9.097s |
| dDocent 2.2.20 | 183,763 | 18m9.770s | 200m39.881s | 5m9.222s |
